# Pathogenicity and Side Effect of Indigenous *Beauveria bassiana* on *Coccinella undecimpunctata* and *Hippodamia variegata* (Coleoptera: Coccinellidae)

**DOI:** 10.3390/insects12010042

**Published:** 2021-01-07

**Authors:** Samy Sayed, Sayed-Ashraf Elarrnaouty, Saad AlOtaibi, Mohamed Salah

**Affiliations:** 1 Department of Science and Technology, University College-Ranyah, Taif University, B.O. Box 11099, Taif 21944, Saudi Arabia; 2 Department of Economic Entomology and Pesticides, Faculty of Agriculture, Cairo University, Giza 12613, Egypt; ashrafelarnaouty@agr.cu.edu.eg; 3 Department of Biotechnology, Faculty of Science, Taif University, B.O. Box 11099, Taif 21944, Saudi Arabia; dralotaibisaad@gmail.com; 4 Department of Biology, Turabah University College, Taif University, B.O. Box 11099, Taif 21944, Saudi Arabia; m.salahh@tu.edu.sa; 5 Zoology and Entomology Department, Faculty of Science, Helwan University, Helwan 11795, Egypt

**Keywords:** compatibility, pathogenicity, entomopathogenic fungi, predators, ladybirds, integrated pest management programs

## Abstract

**Simple Summary:**

Integrated pest management should use integrated or compatible control agents. Most of chemical pesticides have a negative effect on the natural enemies and beneficial microorganisms. Compatible bioagents as entomopathogenic fungi with the natural enemies could achieve higher control impact. Therefore, it is important to test the safety of entomopathogenic microorganisms on the entomophagous insects. The predatory coccinellids are the most effective predatory insect species where attack various insect pests and play very important role in biological pest control in different ecosystems. From these, *Coccinella undecimpunctata* and *Hippodamia variegata* are effective predators of various insect pests and widely distributed in different regions of Saudi Arabia. The present study aimed to estimate the pathogenicity of an indigenous *Beauveria bassiana* isolate on all developmental stages of the abovementioned predators. The obtained results generally showed there were no significant effects on both predator’s mortality and most biological parameters as indirect assay also were not affected as survival, duration, adult longevity, and fecundity.

**Abstract:**

This study aimed to estimate the virulence of an indigenous *Beauveria bassiana* on all developmental stages of two indigenous coccinellids; *Coccinella undecimpunctata* and *Hippodamia variegata* through three application methods; direct spray, contact method, and feeding on aphids treated with the fungus (ingestion). Also, indirect effect on all developmental stages resulted from 1st larval instar treated with these application methods. All treatments were done with a concentration of 1 × 10^5^ which was recommended in previous studies for different aphid species with a control of 0.02% Tween 80 (*v*/*v*). The mortality of 1st larval instar of both *H. variegata* and *C. undecimpunctata* and pupal stage of *C. undecimpunctata* were significantly increased with spray method only. Also, contact method achieved significantly higher mortality on 1st larval instar of *C. undecimpunctata* only. Regard to indirect effect, except of mortality of 1st larval instar of both predators and 2nd larval instar of *H. variegata,* other developmental instars/stages of both predators were not affected by *B. bassiana* through the three tested application methods in the mortality, duration, survival, cumulative survival male and female longevity, and fecundity. Therefore, both tested predatory coccinellids could be compatible with this indigenous isolate of *B. bassiana* where, in general, there are no negative effects of the fungus on both predators.

## 1. Introduction

The chemical pesticides are still a priority in pest control, allowing growers to produce crops of sufficient quality at low costs [1]. In contrast, most chemical pesticides have a negative impact on the natural enemies. Thus, there is need to select safe entomopathogenic microorganisms that could be compatible with entomophagous insects [2].

The predatory insects belonging to family Coccinellidae (Order: Coleoptera) include important natural enemies of several phytophagous pests. They attack various insect pests as aphids, whiteflies, thrips, jassids, mealybugs, lepidopterous eggs, and larvae as well as other small insect pests. They play an important role in biological control of insect pests attacking various plant species [3,4]. The predatory coccinellids, *Coccinella undecimpunctata* L., and *Hippodamia variegata* Goeze (Coleoptera: Coccinellidae) are effective predators of various insect pests such as aphids either in greenhouses or fields due to their high feeding capacity [5,6]. Both of *C. undecimpunctata* and *H. variegata* are widely distributed in different regions of Saudi Arabia [7,8,9].

Entomopathogenic fungi (EPF) often contribute the natural enemies such as predatory coccinellids to reduce the population of herbivorous insects in different ecosystems [10]. On the other hand, some of these EPF such as *Beauveria bassiana* (Balsamo) Vuillemin (Ascomycota: Hypocreales) are generalist EPF infecting phytophagous insects and predatory coccinellids [11]. *B. bassiana* has many positive attributes such as potential infection of up to 80% of pest populations, high genetic variation among various isolates, great diversity, potential mortality of all stages of the targeted pest, high capacity for vertical and horizontal dispersal, and without significant environmental implications [12,13]. 

The field monitoring of some insect predators included *C. undecimpunctata* indicated that *B. bassiana* failed to differentiate between these predators and pests that present in the same environment [14]. Studies on the interaction of many predatory coccinellids and their pathogens had less attention than the pathogens of insect pests [10]. Use of EPF in pest control programs is often based on authorized isolates rather than exotic fungal pathogens [15,16]. The impact of natural EPF attacking predatory coccinellids in the field indicated that natural infection rates were less than 20% [14,17]. *B. bassiana* with high inoculum densities cause mortality to adults of *Coccinella septempunctata*, while mortality could be achieved after field application with extended contact with spores during the mobility on leaves surface [18]. Also, different isolates of *B. bassiana* had different pathogenicity rates on the same species such as *C. maculata* [19]. The predatory coccinellids mortality in its native ecosystem due to naturally infection by *Beauveria* spp. did not regulate the populations [20]. The interactions of host-predator-entomopathogen in agricultural ecosystems may be antagonistically harmful or synergistically to non-targeted insects and beneficial arthropods [11,21]. Thus, the effective application of *B. bassiana* to control of targeted pest depends on high effect against pests and also low pathogenicity against non-target insects, especially natural enemies.

Spray applications of EPF as *B. bassiana* on plants targeted insect pests as aphids would likely expose these pests and other beneficial insects such as predators to this fungus through different ways as direct spray, contact or predation the infected prey. Due to the high importance of predatory coccinellids in biological aphid control, it is important to determine the influence of other biological agents on them, such as insect pathogens and parasitoids. In this study, we examined the effect of indigenous *B. bassiana* on all instars/stages of *C. undecimpunctata* and *H. variegata* through three different application methods; direct spray, contact and feeding on treated prey (ingestion). Moreover, the treated coccinellids at 1st larval instars were investigated till the adult death to determine the side effect on all biological parameters of all the remaining instars/stages. These results could give indications about the compatibility of the using of these bioagents for aphid control in the field.

## 2. Materials and Methods

### 2.1. Fungus Isolate

The fungus isolate used in this study was an indigenous isolate of *B. bassiana* from Saudi Arabia, which was isolated from Taif region (21°9′ N, 40°35′ E) and identified by morphological and molecular tools (Accession numbers: LC338054) [22]. Conidia were propagated on potato dextrose agar (PDA) (Sigma-Aldrich Corp. St. Louis, MO, USA) for 10 days at 25 °C and darkness conditions. Then, conidia were harvested by a cell scraper and placed in 0.02% Tween 80. However, the viability of conidia was estimated before suspension preparation by spreading 1 mL suspension of a 1 × 10^3^ conidia/mL on PDA media in a Petri dish and incubated for 24 h at 25 °C. Then, germinated conidia were estimated by a phase contrast microscope (Olympus, Tokyo, Japan) with 400× of magnification [23]. According to the obtained viability (91%), the concentration of 1.1 × 10^5^ was considered as a final concentration of 1 × 10^5^ conidia/mL.

### 2.2. Insects

Individuals of males and females of coccinellid predators i.e., *H. variegata* and *C. undecimpunctata* were obtained from a rose field in Taif region, Saudi Arabia. Each couple was reared in a cylindrical plastic vessel (10 cm diameter and 8 cm high) with a hole in the lid (4 cm in diameter) and covered by gauze for proper ventilation. They were maintained with *Ephestia kuehniella* Zeller (Lepidoptera: Pyralidae) eggs from Predators Mass Production Laboratory, Faculty of Agriculture, Cairo University, Egypt, and water on a cube of sponge, and their eggs collected daily. Eggs were transferred to plastic Petri dishes, and also larvae were reared in the same dishes till pupation. The rearing was continued for 3 generations where instars/stages of 4th generation were used in the experiments. 

Adult individuals of rose aphid, *Macrosiphum rosae* L. (Hemiptera: Aphididae) were used for ingestion experiment as prey. Infested leaves of rose plants with this aphid were collected and transferred to the laboratory on the same day of the experiment [24].

### 2.3. Bioassay on All Developmental Instars/Stages

The virulence of *B. bassiana* for eggs, larvae, pupae and adults of both coccinellid predators was estimated by three different application methods; i.e., spray, contact and ingestion of prey. A concentration of 1 × 10^5^ conidia/mL (with 0.02% of Tween 80) was used for all bioassay methods. This concentration was recommended for control of *M. rosae* [24] and grapevine aphid [25]. The control individuals (20 for each replicate) were treated with 1 mL of 0.02% of Tween 80 (*v*/*v*). Spraying of fungus suspension or Tween 80 (0.02%) was done by Bürkle spray bottle w/hand pump, 250 mL purchased from Thomas Scientific (Swedesboro, NJ, USA).

#### 2.3.1. Spray

Eggs, all four larval instars, pupa, and adult males and females of both coccinellid species were treated with spray application. Each 20 individuals (1 day old or less, i.e., emerged or moulted in the 24 h before the experiment) as one replicate except eggs were placed in a Petri dish and sprayed with 1.2 mL suspension of 1 × 10^5^ conidia/mL. Each instar/stage treatment or control consisted of 3 replicates except eggs where 100 eggs were treated as only one replicate. After treatment, all instar/stages (except eggs) were kept individually in a Petri dish with sufficient number of *E. kuehniella* eggs. The mortality was recorded daily till 5th day after treatment. In order to confirm fungal infection, dead individuals were kept in an environmental chamber (Barnstead International, model No: 845, Dubuque, IA, USA) with controlled conditions of 26 ± 1 °C, 14 Light: 10 dark (L:D) and 80 ± 5% relative humidity (RH), and observed till whether appearance of mycosis signs or for 10 days after treatment.

#### 2.3.2. Contact

All four larval instars and adult males and females of both coccinellid species were treated with contact method. A filter paper was placed in each Petri dish and 1.2 mL of fungus suspension was sprayed on the filter paper. The Petri dishes with filter papers were left to dry for 1 h. Then, each individual was placed in a Petri dish with sufficient number of *E. kuehniella* eggs and investigated for five days for mortality. Three replicates, each one consisting of 20 individuals, were used for each treatment and control. Dead individuals were kept as previously mentioned.

#### 2.3.3. Ingestion

For the contact method, all four larval instars and adult males and females of both coccinellid species were maintained with aphid nymphs of *M. rosae* those were treated by spray method on the same day. Twenty aphid nymphs on 2–3 leaves of rose plant were placed in a Petri dish and then sprayed with the fungus suspension. After 1 h, the aphid nymphs were transferred to another Petri dish with an individual of predator for 24 h. After this period, the predator was transferred to new petri dish with sufficient number of *E. kuehniella* eggs for five days and investigated for mortality. In the control, aphids were treated with 1.2 mL of 0.02% of Tween 80 (*v*/*v*). Three replicates (20 individuals for each) were used for each treatment and control. Dead individuals were kept as previously mentioned.

### 2.4. Side Effect on Predators through Infected Prey

To study the side effect of *B. bassiana* on both predators, the previous techniques of spray, contact, and ingestion methods was carried out with 30 individuals of newly 1st larval instar of both predators. Also, 30 individuals of non-treated newly 1st larval instar of both predators were used as control. After these treatments, all treated and non-treated individuals were kept individually in a Petri dish with *E. kuehniella* eggs as prey. All individuals were investigated daily till adult emergence to estimate durations and mortality. After adult emergence, each couple was provided with *E. kuehniella* eggs as prey and kept in a cylindrical plastic vessel with a hole in the lid (4 cm in diameter) and covered by gauze for proper ventilation. Adults were investigated daily till death for estimation of longevity and fecundity.

All experiments were done under laboratory conditions of 26 ± 2 °C, 65 ± 8% RH and 14:10 h (L:D). This low humidity was used because it is relatively compatible with the environmental conditions at Taif, Saudi Arabia where the humidity in Taif is ranged from 30% to 68% at day and from 41% to 78% at night, according to The General Authority of Meteorology and Environmental Protection of Saudi Arabia.

### 2.5. Statistics

Prior to data analysis, the homogeneity of the variances was subsequently tested by Levene’s test by Statistical Package for Social Sciences (SPSS) program (IBM Corp., Chicago, IL, USA). One-way analysis of variance (ANOVA) was conducted for experiments contained three treatment groups beside the control; i.e., larval mortality, larval duration, pupal duration, and all adult parameters. Means were compared by the Duncan test (α = 5%). Chi-square test (*X*^2^-test) was used to compare both hatchability and cumulative survival rates for those that had one value for each treatment or control. Meanwhile, *t*-test was used for each predator species to compare between two groups only; i.e., controls and spray treatments on pupa.

## 3. Results

### 3.1. Direct Effects on H. variegata

**Figure 1:**
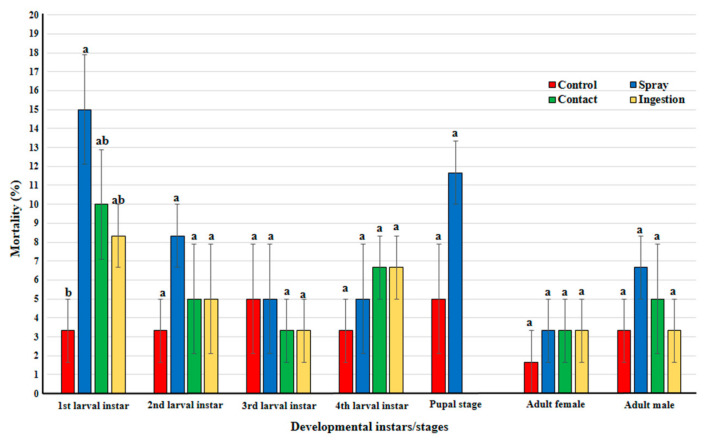
Percent mortality of larva, pupa, and adults of *Hippodamia variegata* treated with three different applications with *Beauveria bassiana.* Different letters above bars indicate significantly different means according to Duncan test (α = 5%) except of pupal stage with *t*-test (α = 5%).

In all developmental stages/instars, except of the 1st larval instar, the mortality rates were not significantly affected by application methods of *B. bassiana* (L_2_; *F* = 0.792; df = 3, 8; *p* = 0.532, L_3_; *F* = 0.167; df = 3, 8; *p* = 0.916, L_4_; *F =* 0.611; df = 3, 8; *p* = 0.627, pupa; *t* = 2.0, df *=* 4, *p* = 0.116, adult female; *F* = 0.25; df = 3, 8; *p* = 0.859, adult male; *F* = 0.611; df = 3, 8; *p* = 0.627). On the other hand, the mortality of 1st larval instar was significantly different (*F* = 4.167; df = 3, 8; *p* = 0.047) where the mortality from the spray method (15%) only was significantly different from that of the control (3.33%) and both of them were not significantly different from those of contact (10%) and ingestion (8.33%) methods (Figure 1). Regard to egg stage treated with the spray method, there was no significant effect (*X*^2^ = 0.139, df = 1, *X*^2^_0.05, 1_ = 3.841, *p* = 0.71) on hatching rate between treated (90%) and non-treated (86%) eggs.

### 3.2. Direct Effects on C. undecimpunctata

**Figure 2:**
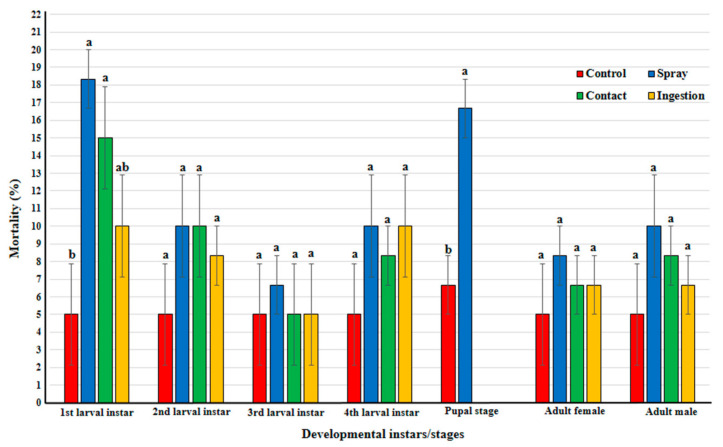
Percent mortality of larva, pupa, and adults of *Coccinella undecimpunctata* treated with three different applications with *Beauveria bassiana.* Different letters above bars indicate significantly different means according to Duncan test (α = 5%) except of pupal stage with *t*-test (α = 5%).

The mortality rates of 1st larval instar were significantly affected by application methods of *B. bassiana* (*F* = 4.9; df = 3, 8; *p* = 0.032) where the larval mortality from both spray (18.33%) and contact (10%) methods was significantly different from that of the control (3.33%) (Figure 2). Meanwhile, mortality from ingestion method (10%) did not differ significantly compared to other treatments and the control (Figure 2). Also, for the pupal stage, the spray method achieved significantly higher mortality (16.67%) compared to the control (6.67%) (*t* = 4.243, df = 4, *p* = 0.013). In contrast, the mortality rates of other developmental stages/instars were not significantly affected by application methods of *B. bassiana* (L_2_; *F* = 0.8; df = 3, 8; *p* = 0.528, L_3_; *F* = 0.1; df = 3, 8; *p* = 0.958, L_4_; *F* = 0.8; df = 3, 8; *p* = 0.528, adult female; *F* = 0.44; df = 3, 8; *p* = 0.728 and adult male; *F* = 0.833; df = 3, 8; *p* = 0.512). Hatchability of egg stage treated with the spray method (85%) did not differ significantly from the control (87%) (*X*^2^ = 0.047, df = 1, *X*^2^_0.05, 1_ = 3.841, *p* = 0.828).

### 3.3. Side Effect on H. variegata through Infected Prey

The 1st larval instar duration of *H. variegata* were significantly increased with both spray and ingestion methods (3 and 3.04 days, respectively) compared to those with both contact method and the control (2.68 and 2.57 days, respectively) (Table 1). Meanwhile, 2nd larval instar duration was significantly prolonged by ingestion application (2.73 days) only more than that of the control (2.33 days). The durations of other developmental larval instars and pupa were not affected by all three application methods. However, durations from 1st larval instar to pupae with both spray (14.71 days) and ingestion (14.88 days) methods were significantly increased than that of the control (14.11 days) but the contact method (14.15 days) did not differ significantly from that of the control.

The survival for each individual instar/stage and accumulated survival (from 1st larval instar to adult) were not significantly different among all treatments and the control (Table 1) being accumulated survival between 80 and 90%.

There were no effects of *B. bassiana* on adult stage treated at their 1st larval instar with three different application methods of the fungus (Table 2) where male and female longevity and fecundity not affected by these fungus applications.

### 3.4. Side Effect on C. undecimpunctata through Infected Prey

Exactly as in *H. variegata*, the durations of 1st larval instar of *C. undecimpunctata* treated with the three different application methods by *B. bassiana* suspension were significantly different. This duration was significantly prolonged with both spray (3.11 days) and ingestion (3.19 days) methods than those by both contact method (2.71 days) and the control (2.79 days) (Table 1). In contrast with *H. variegata*, 2nd larval instar durations of *C. undecimpunctata* not significantly affected by all treatments compared to the control (2.63–2.88 days). In the same context of *H. variegata,* the durations of other developmental larval instars and pupa, their survival, male and female longevity, and fecundity (Table 1 and Table 2) were not affected by all three application methods. Meanwhile, durations from 1st larval instar to pupae were not significantly different among treatments and the control (Table 1).

## 4. Discussion

Information that shows the susceptibility of indigenous species of coccinellids to indigenous EPF is very important for using them in a combination in pest control programs [11]. Selection of EPF is important in their use in pest control programs to obtain effective pest control of agricultural crops [26]. Compatible bio-products as entomopathogenic fungi with the natural enemies could achieve higher control impact with decreasing the use of conventional insecticides, minimizing hazards of ecological pollution, and reducing the resistance to insecticide [27]. In the present study, the effects were shown through direct spray, contact method or by feeding on aphids treated with the fungus on the mortality rates of both tested coccinellids. The mortality of 1st larval instar of both *H. variegata* and *C. undecimpunctata* and pupal stage of *C. undecimpunctata* were significantly increased with spray method only. Also, contact method achieved significantly higher mortality on 1st larval instar of *C. undecimpunctata* only. Other developmental stages of *H. variegata* and *C. undecimpunctata* were not affected by the tested isolate of *B. bassiana* through the three tested application methods. In this context, B. bassiana achieved the highest mortality rate on larval stage of *Cryptolaemus montrouzieri* Mulsant (Coleoptera: Coccinellidae) than other developmental stages [28]. Similarly, different laboratory investigations indicated that application of recommended concentrations of commercial *B. bassiana* is compatible with beneficial insects. For examples, *B. bassinna* was not pathogenic to different natural enemies such as *C. septempunctata* [29]. The preimaginal stages of *Chrysoperla exotera* (Navás) (Neuroptera: Chrysopidae) were not affected when treated with high concentration of *B. bassiana* [30].

Our results indicated that both tested coccinellids were not affected in their mortality with feeding on aphid treated with the fungus weather for the same coccinellid instar/stage or for instar/stage resulted from treated 1st larval instar. In this context, *Coleomegilla maculata* fed on Colorado potato beetle *Leptinotarsa decemlineata* Say (Coleoptera: Chrysomelidae) infected with *B. bassiana* and contaminated pollen with *B. bassiana* resulting in no mortality of *C. maculata* [31]. This investigation indicated that *H. variegata* was less susceptible than *C. undecimpunctata* in their 1st larval instar and pupa. In other way, *H. variegata* in alternative option indicated a significant attraction towards aphids non-infected than aphids infected by *B. bassiana* from 1 to 3 days after treatments with a concentration of 1 × 10^5^ conidia/mL. This suggested that *H. variegata* could avoid *B. bassiana* under laboratory conditions. Therefore, this fungus was a negligible threat to this predator [32]. Our results showed that there was no effect on most instar/stages of both coccinellids through the different application methods. Moreover, results showed that the cumulative survival of both coccinellids were not affected with all treatments compared to the control. An investigation of the impact of *B. bassiana*, *Metarhizium rileyi* and *Metarhizium anisopliae* on *Chrysoperla externa* (Hagen) indicated that there were no differences among the application method or concentration of conidia. Also, no larval mortality of *C. externa* above 3% at 4 days after treatments. Meanwhile, after 5 days, *B. bassiana* only achieved significant mortality in all larval instars (26% in L1, 17% in L2 and 10% in L3) [33].

Generally, the same fungus isolate could have diverse effects on different hosts, even if they are from the same family. The American authorized strain NI8 *B. bassiana,* which was isolated from tarnished plant bug, *Lygus lineolaris,* can infect various insect predators including *H. axyridis,* but with LC_50_ values of 3- to 90-fold those of *L. lineolaris* [34]. In contrast, the same isolate had significant effects on adults of *Chrysoperla rufilabris* [35] and low effects on *H. axiridis*, *Orius insidiosus*, *H. convergens* and *C. maculata* [36]. Moreover, there are differential susceptibility among coccinellid species to EPF. For example, *Harmonia axyridis* and *Olla v-nigrum* (Coleoptera: Coccinellidae) were collected from the same habitats indicated that *O. v-nigrum* had low mortality and none of *H. axyridis* were infected with *B. bassiana* [37]. This indicated the possibility of different susceptibility between the indigenous *O. v-nigrum* and the introduced *H. axyridis*. Moreover, *B. bassiana* that isolated from naturally infected *O. v-nigrum* was not pathogenic with a concentration of 2.5 × 10^5^ conidia/mL to the introduced *H. axyridis* and the indigenous *Coleomegilla maculata* but pathogenic to the indigenous species *O. v-nigrum*, *Hippodamia convergens* and *Cycloneda munda*.

Regard to the side effect on treated 1st larval instar and their other larval instars, pupa, and adults. Both spray and ingestion treatments had a significant increase in 1st larval duration of both coccinellids but only ingestion treatment had a significant increase in 2nd larval duration of *H. variegata*. Similarly, the 4th larval instar of *C. undecimpunctata* when fed on aphid treated with *B. bassiana* (Biosect) did not affected in its duration but duration of its pupae was significantly decreased than that of the control [38].

All treatments in this study did not significantly affect the male and female longevity and fecundity of both coccinellid species. In other study, adults fed on aphid treated with a low concentration (0.5 g/L) of *B. bassiana* was not affected in the male and female longevity but fecundity was significantly lower than that of the control [38]. Also, both female and male of *C. septempunctata* were influenced similarly by *B. bassiana* [39].

## 5. Conclusions

Both tested coccinellid predators, in general, were not affected by *B. bassiana* through the three tested application methods in the mortality, duration, survival, cumulative survival, adult longevity, and fecundity. Therefore, both tested predatory coccinellids could be used in association with this isolate of *B. bassiana* for sustainable pest management.

## Figures and Tables

**Table 1:** Effect of *Beauveria bassiana* on the developmental time (days ± standard error) and cumulative survival (%) of larva, pupa, and adults of *Hippodamia variegata* and *Coccinella undecimpunctata* treated at their 1st larval instar with three different application methods of the fungus.

Application Method	1st Larval Instar		2nd Larval Instar		3rd Larval Instar		4th Larval Instar		Pupa		Total(Larva-Adult)	
	Duration	Survival	Duration	Survival	Duration	Survival	Duration	Survival	Duration	Survival	Duration	Survival
***Hippodamia variegata***												
**Spray**	3.00 ± 0.11 a	90.00 a	2.60 ± 0.10 ab	83.33 a	2.28 ± 0.09 a	83.33 a	3.33 ± 0.10 a	80.00 a	3.54 ± 0.11 a	80.00 a	14.71 ± 0.18 ab	80.00 a
**Contact**	2.68 ± 0.09 b	93.33 a	2.56 ± 0.10 ab	90.00 a	2.23 ± 0.08 a	86.67 a	3.23 ± 0.08 a	86.67 a	3.50 ± 0.10 a	86.67 a	14.15 ± 0.22 bc	86.67 a
**Ingestion**	3.04 ± 0.10 a	93.33 a	2.73 ± 0.09 a	86.67 a	2.32 ± 0.10 a	83.33 a	3.21 ± 0.10 a	80.00 a	3.58 ± 0.10 a	80.00 a	14.88 ± 0.20 a	80.00 a
**Control**	2.57 ± 0.10 b	93.33 a	2.33 ± 0.09 b	90.00 a	2.33 ± 0.10 a	90.00 a	3.30 ± 0.13 a	90.00 a	3.56 ± 0.10 a	90.00 a	14.11 ± 0.19 c	90.00 a
***F* (df)**	5.71 (3107)		3.09 (3101)		2.61 (3.99)		2.88 (3.97)		0.12 (3.97)		3.83 (3.97)	
***X*^2^ (df = 3)**		0.089		0.349		0.356		0.891		0.891		0.891
***p***	0.001	0.993	0.031	0.951	0.853	0.949	0.834	0.827	0.949	0.827	0.012	0.827
***Coccinella undecimpunctata***												
**Spray**	3.11 ± 0.11 a	90.00 a	2.74 ± 0.11 a	76.67 a	2.52 ± 0.13 a	76.67 a	3.55 ± 0.13 a	73.33 a	3.65 ± 0.17 a	66.67 a	15.50 ± 0.36 a	66.67 a
**Contact**	2.71 ± 0.09 b	93.33 a	2.76 ± 0.09 a	83.33 a	2.54 ± 0.10 a	80.00 a	3.46 ± 0.10 a	80.00 a	3.73 ± 0.19 a	73.33 a	15.32 ± 0.27 a	73.33 a
**Ingestion**	3.19 ± 0.11 a	90.00 a	2.88 ± 0.11 a	90.00 a	2.67 ± 0.13 a	80.00 a	3.39 ± 0.10 a	76.67 a	4.00 ± 0.17 a	76.67 a	16.09 ± 0.36 a	76.67 a
**Control**	2.79 ± 0.08 b	93.33 a	2.63 ± 0.10 a	90.00 a	2.69 ± 0.11 a	86.67 a	3.62 ± 0.16 a	86.67 a	4.04 ± 0.21 a	83.33 a	15.76 ± 0.30 a	83.33 a
***F* (df)**	5.86 (3106)		1.09 (3.96)		0.55 (3.93)		0.61 (3.91)		1.10 (3.86)		1.08 (3.86)	
***X*^2^ (df = 3)**		0.123		1.066		0.653		1.229		1.925		1.925
***p***	<0.001	0.989	0.355	0.785	0.648	0.884	0.612	0.746	0.354	0.588	0.365	0.588

For each coccinellid species, means within each column bearing different letters are significantly different according to Duncan test (α = 5%).

**Table 2:** Effect of *Beauveria bassiana* on male longevity, preoviposition, oviposition, postoviposition, total female longevity (days ± standard error) and fecundity (eggs/female) of *Hippodamia variegata* and *Coccinella undecimpunctata* treated at their 1st larval instar with three different application methods of the fungus.

Application Method	Tested Individuals	Male Longevity	Pre-Oviposition	Oviposition	Post-Oviposition	Female Longevity	Fecundity
***Hippodamia variegata***							
**Spray**	**9**	42.78 ± 3.87	4.11 ± 0.31	37.22 ± 3.07	4.33 ± 1.05	45.56 ± 4.03	904.3 ± 66.7
**Contact**	**10**	40.60 ± 3.57	3.70 ± 0.26	37.60 ± 2.33	3.10 ± 0.78	43.90 ± 2.58	828.3 ± 65.8
**Ingestion**	**9**	42.11 ± 3.59	3.67 ± 0.33	40.56 ± 2.49	4.67 ± 0.97	48.67 ± 3.45	858.6 ± 68.2
**Control**	**10**	43.90 ± 3.50	3.90 ± 0.32	43.50 ± 3.80	4.30 ± 0.91	50.00 ± 4.35	929.3 ± 67.9
***F* (df = 3.34)**		0.151	0.441	0.974	0.563	0.601	0.470
***p***		0.928	0.725	0.417	0.643	0.619	0.705
***Coccinella undecimpunctata***							
**Spray**	**8**	37.88 ± 4.54	4.25 ± 0.41	40.00 ± 5.21	2.13 ± 0.83	46.38 ± 5.89	1001.3 ± 112.5
**Contact**	**10**	42.60 ± 4.70	4.30 ± 0.30	43.10 ± 4.67	2.90 ± 0.59	50.30 ± 4.99	941.3 ± 98.6
**Ingestion**	**9**	44.11 ± 5.00	4.56 ± 0.38	40.89 ± 4.73	4.33 ± 0.94	49.78 ± 5.35	1021.1 ± 113.0
**Control**	**10**	41.10 ± 4.95	5.10 ± 0.38	45.90 ± 4.41	3.50 ± 0.83	54.50 ± 4.88	1082.6 ± 93.1
***F* (df = 3.33)**		0.278	1.18	0.308	1.263	0.395	0.340
***p***		0.841	0.333	0.820	0.303	0.757	0.796

In the same species, means within each column are not significantly different according to Duncan test (α = 5%).

## Data Availability

Data is contained within the article.
